# Experimental Investigations on Electric-Field-Induced Crystallization in Erythritol

**DOI:** 10.3390/ma14175110

**Published:** 2021-09-06

**Authors:** Jean-Luc Dauvergne, Artem Nikulin, Stefania Doppiu, Elena Palomo del Barrio

**Affiliations:** 1 Centre for Cooperative Research on Alternative Energies (CIC energiGUNE), Basque Research and Technology Alliance (BRTA), Alava Technology Park, Albert Einstein 48, 01510 Vitoria-Gasteiz, Spain; anikulin@cicenergigune.com (A.N.); sdoppiu@cicenergigune.com (S.D.); epalomo@cicenergigune.com (E.P.d.B.); 2 Ikerbasque, Basque Foundation for Science, 48013 Bilbao, Spain

**Keywords:** electric field, supercooling, crystallization, sugar alcohol, erythritol

## Abstract

The objective of this experimental study was to develop a method to induce crystallization of sugar alcohols using an electric field for its future implementation in latent heat thermal energy storage systems. To better understand the mechanisms behind this approach, the first step of this work was dedicated to the replication, continuation, and consolidation of promising results on erythritol reported by another research group. In the second step, a second experimental configuration, previously used to electrically control the supercooling of other phase change materials, was tested with the same sugar alcohol. For both configurations, the influence of the type of current (DC and AC at different frequencies), its amplitude, and time of exposure were studied. However, none of these tests allowed influencing the crystallization of erythritol. Even if surprising at first glance, the difficulty in reproducing experiments and interpreting the results is not new in the field of electric-field-induced crystallization, as shown in particular by the abundant literature reviews concerning water. Currently, to the best of our knowledge, we consider that electric fields could be an attractive option to initiate and accelerate the crystallization of erythritol, but this solution must be considered with caution.

## 1. Introduction

The depletion of energy resources and human-induced climate change are challenging the sustainability of our societies and make the need to promote renewable energies and energy efficiency undeniable. According to Forman et al. [1] and a report of the US Department of Energy [2], 72% of the global primary energy consumption is lost after conversion and 79% of the overall heat waste takes place below 300 °C and 60% below 230 °C. In particular, in the industrial sector, about 60% of the heat losses occur in this temperature range. Each industry has its own production cycles where heat demand and heat surplus are scattered across time. This fact makes heat recovery difficult since heat demand and surplus should be coupled in time and cannot be simply recovered by heat exchanger networks. This coupling can be achieved by integrating thermal energy storage (TES) systems for waste heat recovery.

Between the different categories of TES (sensible, latent, and thermochemical), latent heat thermal energy storage (LHTES) offers a good compromise between high heat storage density and reasonable simplicity of system handling and operation. LHTES relies on a storage material that absorbs or releases heat as it undergoes a phase transition, commonly a solid–liquid phase change.

Among the available phase change materials (PCMs), polyols (or sugar alcohols) constitute a group with the most outstanding characteristics (high mass-specific and volume-specific enthalpies, good sustainability, reasonable prices, and few chemical interaction with metals [3]) and are among the few that melt at the desired temperature for low-temperature heat recovery (80 to 230 °C) [4,5]. However, one important aspect requires further research and engineering to enable LHTES systems with polyols as PCMs, namely the control and management of the discharge stage. Thermal discharge of the system requires crystallization of the molten polyol. Nonetheless, at normal operational cooling rates, most polyols tend to vitrify instead of crystallizing. Additionally, when crystallization takes place, it usually happens at low rates, which limits the discharging power obtained. To overcome such issues, different methods to crystallize polyols from their melt have been studied during the last years: seeding, mechanical stirring, local cooling, additives, and ultrasound-assisted crystallization [6,7]. However, those that have only local effects make them inefficient to counterbalance the slow crystal growth of many polyols. On the contrary, stirring by bubbling, in which small bubbles distributed throughout the melt act as effective nucleation sites, overcomes such drawbacks [8].

An appealing alternative to the previous approaches could be electric-field-driven crystallization of polyols, mainly because integrating one or several sets of electrodes in LHTES should be easier than integrating, for example, an ultrasonic or a mechanical stirring setup. 

The effect of electric fields (E-fields) on polar molecules and hydrogen bond networks has been studied for a long time. The most studied case is that of water, for which there is an abundance of experimental and theoretical studies [9,10]. It is known that E-fields can orient water molecules and change O–H bond characteristics (length, bond angles, vibrational energy, etc.). Depending on its strength, the E-field can also lead to the disruption of the H-bond network and even provoke water dissociation. Despite substantial progress, the mechanism by which the E-field interacts with water remains a widely debated research topic [11]. However, there is experimental evidence that the application of E-fields between 0.45 V/m and 3 GV/m encourages ice formation in supercooled water and accelerates its crystallization [12,13].

The use of E-fields to crystallize proteins, which contain different ionic and polar groups, is also under investigation. Since the seminal work of [14], electric-field-induced crystallization of proteins from solutions has been studied by many other authors [10,15,16]. They have proved that this technique can be applied to the crystallization of proteins that have so far resisted crystallization by conventional methods. It is believed that the three main effects of E-fields on crystal nucleation are the alignment of molecules, the modification of the nucleation work, and the induction of concentration gradients.

E-field-induced crystallization from solutions has also been proposed for pharmaceutical industry applications. It has been evidenced that the E-field not only promotes nucleation but also is a way for controlling the polymorphic outcome of a cooling crystallization process [17].

The scarce research performed on the use of electric fields for the crystallization of polyols as PCMs has focused on erythritol [18]. Experimental tests performed showed a positive behavior of this material. However, further research is needed to understand this technique, to extend it to polyols that exhibit a slow recrystallization rate, and to develop a mechanism that allows its implementation on LHTES systems.

According to this last remark, the objectives of this study were (i) the pursuit and consolidation of this previous work, (ii) the understanding of the mechanisms of E-field-induced crystallization with sugar alcohols, and (iii) the development of a suitable method that allows their future use in LHTES systems.

## 2. Materials and Methods

### 2.1. Materials

Erythritol (99%) (Alfa Aesar, Kandel, Germany) was used as received. The main properties of this sugar alcohol are shown in Table 1.

**Figure 1:**
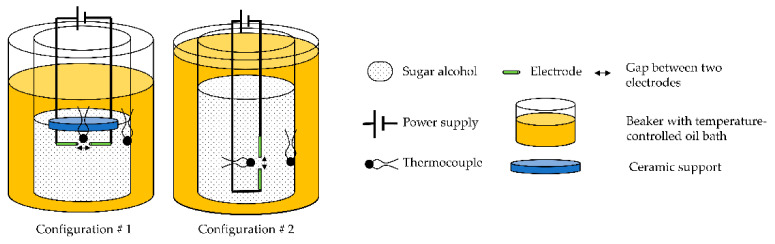
Sketch of the two configurations tested in this work.

To study the impact of an electric field on the nucleation of erythritol, two configurations, inspired by previous studies, were tested in this work (in Figure 1). Both are detailed in the Methods section. They consist of introducing two electrodes, connected to a power supply, and two thermocouples into molten erythritol at a temperature below its melting point (supercooled state).

The different electrodes used in the two configurations are presented in Table 2.

Gaps between two electrodes were estimated by simple data processing (pixel counting) from zoomed images showing the electrodes and a reference of known dimensions.

Different power supplies were used to generate a potential difference between the electrodes: a Chroma 61605 allowed us to work in DC (up to 212 V/424 V for 16 A/8 A respectively, up to 2 kW) and AC (up to 4000 VA, 15 to 1000 Hz). An EA-PSI 9500-10 T was also used to perform additional tests in DC (up to 500 V and 10 A, up to 1.5 kW). Finally, a piezoelectric element was used to generate a short (ca. 0.05 s) high-voltage (ca. 15 kV) discharge.

In addition, a manual switch was added between the power supply and the positive electrode for the possibility to interrupt the electrical input without the latter discharging to the power supply ground (“floating electrode”). Finally, ammeters (Fluke 233 and Fluke 116, maximum 10 A ± 1.5% and 600 µA ± 1.5%, respectively) were used to monitor the current intensity during the experiments.

To melt and maintain the sugar alcohol at the desired temperature, a temperature-controlled hot plate with a magnetic stirrer (Bibby Scientific, Nemours, France) (Stuart UC152) was used to regulate the temperature of a silicone oil bath (regulation at ±0.5 °C around the desired temperature).

Temperature measurements and thermal regulation of the oil bath were ensured using type K thermocouples and Arduino Nano coupled with 3 MAX 31856 modules (thermocouple amplifier/converter with integrated cold-junction compensation) (Adafruit Industries, LLC, New York, NY, USA). The measurement accuracy of each set of thermocouple/amplifier modules was checked at different temperatures using a temperature-controlled bath (Julabo 1000F) (JULABO GmbH, Seelbach, Germany) and a furnace (Carbolite CWF 1100) (Carbolite Gero GmbH & Co. KG, Neuhausen, Germany) coupled with other temperature measurement systems (National Instruments NI 9211 and Fluke 233) (National Instruments, Austin, TX, USA; Fluke, DC, USA). According to these tests, the accuracy of temperature measurements is less than 2% of a given reading.

Dedicated software for thermocouple acquisition was coded using Python 3.7.3 (Python Software Foundation, Beaverton, OR, USA, Arduino 1.8.13 (Arduino LLC, Somerville, MA, USA), and the Adafruit_MAX31856 library (Adafruit Industries, LLC, New York, NY, USA) [21].

### 2.2. Methods

#### 2.2.1. Thermodynamic Characterization of Erythritol

Differential scanning calorimetry (DSC) was used to verify the transition temperature, as well as the corresponding latent heat of our batch of erythritol. A power-compensation DSC Q2500 (TA Instruments, New Castle, DE, USA) was employed with a closed aluminum crucible. The mass of the sample was ca. 9 mg. Argon (50 mL/min) was used as a purge gas. The temperature and enthalpy were calibrated using sapphire and indium standards: the accuracy was estimated to be ±1 K for temperature and ±0.02 J/g for enthalpy. The sample was subjected to a first heating-cooling cycle (from −20 °C to 160 °C with a heating/cooling rate of 5 K/min) in an oven to ensure the airtightness of the crucible. Then the sample was subjected to the same three cycles of heating and cooling in DSC. The sample was weighed before and after the DSC measurements using a Mettler Toledo high-precision balance (±0.001 mg), and no mass loss or leakage during the analysis was observed.

#### 2.2.2. E-Field-Induced Crystallization

The two configurations and associated experimental protocols are presented in detail in this section. One can note that some steps of the protocols are common whatever the configuration. 

In the first step, the sample of erythritol was melted in a beaker placed in an oil bath and cooled freely to verify that the amount of material tested (5 g in configuration 1 and 15 g in configuration 2) exhibited a similar degree of supercooling to that observed by standard DSC. To perform this first step, one thermocouple was introduced since the beginning in the beaker (called TC2 in the following section).

In the second step, the sugar alcohol was melted again, the set electrodes/thermocouples were introduced in the molten polyol, and then this ensemble was cooled freely to check that the final configuration did not significantly affect the degree of supercooling.

Thereafter, different electric fields were imposed on the molten erythritol in the supercooled state, and their potential impact on its nucleation was studied.

Finally, for both configurations, in the absence of E-field-induced crystallization, the beaker was cooled freely to verify that the tested erythritol crystallized at a similar temperature to that observed during the second step and was not degraded during the test.

#### 2.2.3. Configuration 1

**Figure 2:**
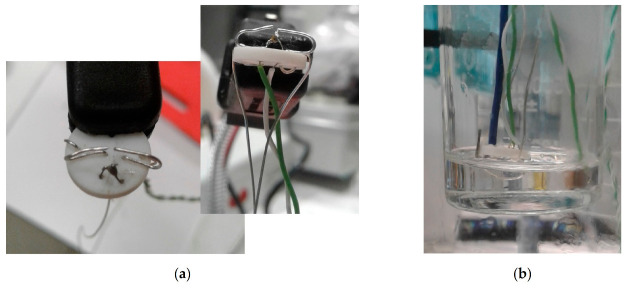
Electrode assembly (**a**) bottom and profile views and (**b**) submerged in the 30 mL beaker.

This configuration consisted of a first stage of reproducing the experimental setup of Jankowski and McCluskey [18], with the aim of checking their promising results with erythritol and continuing their work in the second stage. The device described by the authors and used in this work is as follows (in Figure 2):

- First, 5 g of powdered erythritol was placed in a 30 mL beaker. A unique sample was used for all tests.
- The electrical solicitation system consisted of an assembly of 2 silver electrodes (diameter 375 µm) fixed onto a ceramic support (Macor^®^ glass ceramic, diameter 10 mm, thickness 1 mm). The ends of these electrodes were cut using wire cutters. The space between them was ca. 400 µm.
- The first thermocouple (TC1) was fixed on the ceramic support. Its end was located approximately 1 mm next to the electrodes.
- A second thermocouple (TC2) was also placed inside the beaker in contact with the glass at the same height as the electrodes.
- The beaker was then heated using an oil bath (in a 500 mL beaker) and a hot plate. Homogeneity of the oil temperature (at approximately 140 °C) was ensured by magnetic stirring and controlled using a third thermocouple, called TCbath until the erythritol reached a temperature of about 130 °C. After complete melting of the erythritol, the electrode/thermocouple/ceramic assembly was immersed in the latter, maintaining approximately a 2 mm distance between the electrodes and the bottom of the beaker.
- A power supply was connected to the electrodes. The manual switch was used to stop the electrical solicitation (“floating electrode”).
- Before any electrical solicitation, the erythritol was freely cooled down by removing the beaker from the oil bath. For this purpose, a low-speed stepper motor (−3 mm/s) was added to the original device to extract the beaker, ensuring the repeatability of the experiments, while limiting vibrations during this step.

#### 2.2.4. Configuration 2

**Figure 3:**
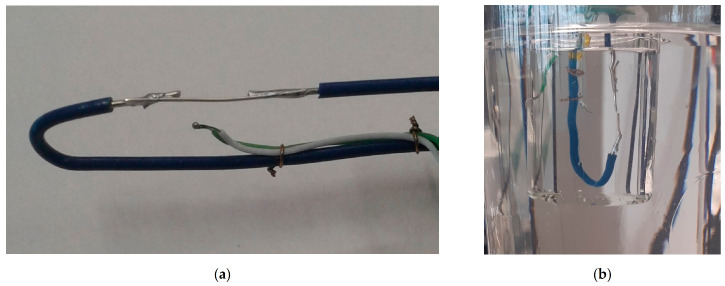
Configuration 2: (**a**) electrode assembly before section of the central silver wire in two electrodes; (**b**) submerged in the 30 ml beaker.

The second configuration was inspired by the works of Hozumi et al. [22,23] and Kumano et al. [24,25] to study the effect of an electric field on supercooled water and tetra-n-butyl ammonium bromide hydrate, respectively. The experimental setup can be described as follows:

- First, 15 g of powdered erythritol was placed in a 30 mL beaker, and a unique sample was used for all tests.
- The electrical solicitation system consisted of an assembly of two electrodes soldered on two rigid wires (in Figure 3). These electrodes were made with silver or copper wires (diameters 375 and 200 µm, respectively). The ends of the electrodes, with a gap of 0.15 mm, were cut using wire cutters.
- TC1 was also fixed on the rigid wires. Its extremity was located at approximately 1 mm from the electrodes. TC2 was placed inside the beaker in contact with the glass at the same height as the first thermocouple (in Figure 1).
- As in configuration 1, the beaker was then heated using the oil bath and the hot plate. The temperature of the oil was maintained at ca. 140 °C (TCbath) until the erythritol had completely melted. Then, the electrode/thermocouple assembly was immersed in the erythritol and connected to a power supply with a manual switch (“floating electrode”). One can note that a configuration without a manual switch, to allow discharge to the ground of the power supply, was also tested during our experiments.
- In this configuration, and before any electrical solicitation, the cooling of the erythritol to a given temperature was ensured by regulating the temperature of the oil bath.

## 3. Results and Discussion

### 3.1. Preliminary Tests

This first step of the two experimental protocols aimed to evaluate the impact of the quantity of erythritol and of the implementation of the electrode’s assembly on the reduction of its supercooling, an essential preliminary step for the subsequent evaluation of the impact of electric fields on the latter.

#### 3.1.1. Thermodynamic Characterization of Erythritol

**Figure 4:**
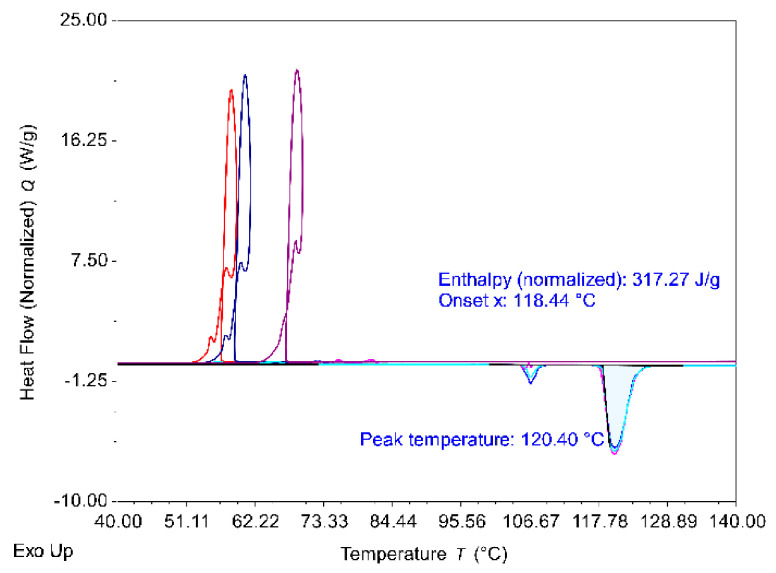
DSC thermograms (3 cycles) obtained in ramp mode at a heating/cooling rate of 5 K/min for erythritol.

Following the procedure described in Section 2.2.1, the batch of erythritol was characterized using standard DSC. The obtained thermograms are depicted in Figure 4, and the estimated properties are summarized in Table 3.

The erythritol used in this work showed properties in agreement with the literature (in Table 1) as well as a comparable degree of supercooling of about 63 °C.

#### 3.1.2. Configuration 1: Without an E-Field

**Figure 5:**
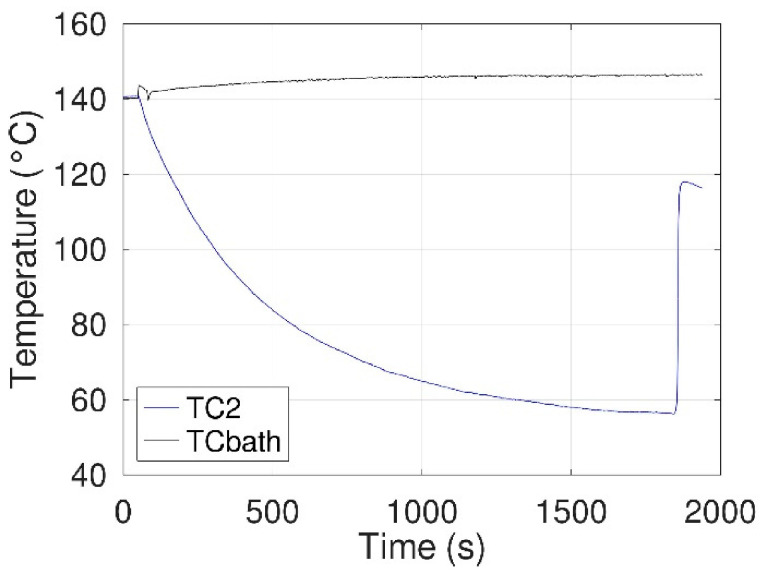
Thermograms (oil bath, TCbath, and erythritol, TC2erythritol, recorded during free cooling without introducing the electrode assembly in the vial.

Before proceeding with the experimental protocol associated with the first configuration (in Section 2.2.2) and detailed in the following section, a preliminary verification was carried out to ensure that the 5 g of erythritol still exhibit a high degree of supercooling in the tested configuration. In this way, a heating-free cooling cycle was performed without introducing the electrode assembly (i.e., electrodes, thermocouple TC1, and ceramic support). As shown in Figure 5, the degree of supercooling was comparable to that observed in standard DSC measurements, i.e., no crystallization was observed up to a temperature of 60 °C below the melting temperature.

In the second step, the sample of erythritol was melted again and the electrode assembly was introduced into the sugar alcohol. After this step, series of voltage-free tests were carried out (tests designated as PRE##) as follows:

1. A 30 mL beaker containing the sample was heated in an oil bath until erythritol melted and thermocouple TC1 indicated a temperature of about 130 °C (~12 °C above the melting temperature of erythritol).
2. The beaker was then removed from the bath, using the low-speed stepper motor, to cool it down by natural convection.
3. Temperatures were measured until crystallization of the erythritol.

In this configuration, Jankowski and McCluskey [18] observed that, as expected, the crystallization of erythritol was initiated at the edge of the beaker, i.e., where the temperature is the lowest. In their experiments, this nucleation temperature averaged 86.2 °C, with variations up to 15 °C (standard deviation of 9.7 °C).

**Figure 6:**
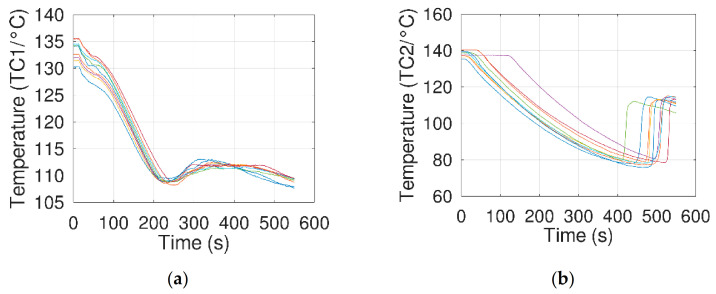
Thermograms obtained during free cooling without an electrical field (PRE01 to PRE09). (**a**) Thermocouple fixed to the electrode assembly (TC1) and (**b**) thermocouple fixed on the internal wall of the beaker (TC2).

The tests carried out in the present work differ radically from those obtained by the authors. Indeed, the temperature profiles obtained during nine tests (in Figure 6) all showed a crystallization of erythritol at the level of the electrode assembly, never at the edge of the vial, with a low degree of supercooling. Thus, erythritol crystallizes at an average temperature of 108.5 °C, with excellent repeatability (standard deviation of 0.5 °C; in Table 4). Surprisingly, these results show comparable reduction in the supercooling with better reproducibility than that obtained by Jankowski and McCluskey when they applied an electrical field to the sample.

The initial nucleation location and the spectacular reduction in the degree of supercooling can be induced by a thermal bridge effect due to the high thermal conductivities of the electrodes/thermocouple wires. This thermal bridge tends to the sample locally below its usual temperature of crystallization, with a consequent reduction in the supercooling at the sample scale.

Despite these first results, a second series of tests was carried out by applying different voltages to the electrodes to assess a possible impact of the latter on the crystallization temperature of erythritol. The corresponding experimental protocol and results are described in Section 3.2.

#### 3.1.3. Configuration 2: Without an E-Field

**Figure 7:**
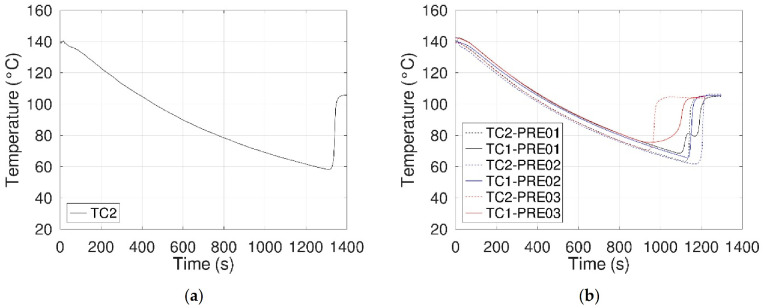
Thermograms obtained during free cooling without an electrical field. (**a**) Without electrode assembly (TC2) and (**b**) with electrode assembly (TC1 and TC2).

For the second configuration and following a protocol like that of the first one, two preliminary tests were performed. In the first step, the sample, without introducing the electrode assembly, was heated up to a temperature of 140 °C and freely cooled down. As shown in Figure 7a, 15 g of erythritol exhibited a similar degree of supercooling to that observed with the previous configuration (5 g; in Figure 5). In the second step, the erythritol was melted again and the electrode assembly was introduced into the sample. In this configuration, three other cycles of heating–free cooling were performed (designated as PRE##). The obtained thermograms and the corresponding temperatures of crystallization (TC1) are presented in Figure 7b and Table 5, respectively. 

Contrary to configuration 1, a high degree of supercooling was observed, allowing us to evaluate the impact of electric fields on the crystallization of erythritol. One can note that crystallization always occurs on the electrode assembly (Table 5), not in a specific point of the latter, at a higher temperature than that of the one observed without introducing the electrode assembly, ca. 70 °C versus 58 °C. As expected, this configuration limits, without elimination, the heat sink effect observed with the first configuration.

### 3.2. E-Field Impact on Erythritol Crystallization

After the preliminary tests reserved for the evaluation of the electrode assembly on the reduction in supercooling of the polyol, this section details the tests carried out under electrical solicitations to assess their impact on its nucleation.

#### 3.2.1. Configuration 1

To assess the possible impact of the E-field on erythritol crystallization, the following experimental protocol was applied with the first configuration:

1. A 30 mL beaker containing the sample was heated in an oil bath until erythritol melted and thermocouple TC1 indicated a temperature of about 130 °C (~12 °C above the melting temperature of erythritol).
2. The sample was then carefully removed from the bath and cool down by natural convection.
3. When TC1 indicated a temperature of 115 °C, a potential difference, from 25 to 200 V, was imposed for 3 s between the electrodes. This electrical stress was then interrupted using the manual switch.
4. Temperatures from thermocouples TC1, TC2, and TCbath were recorded from step 2 until complete crystallization of the erythritol. During this time, the beaker was visually inspected to identify the initial crystallization location.
5. Steps 1 to 4 were repeated for each experiment.

**Figure 8:**
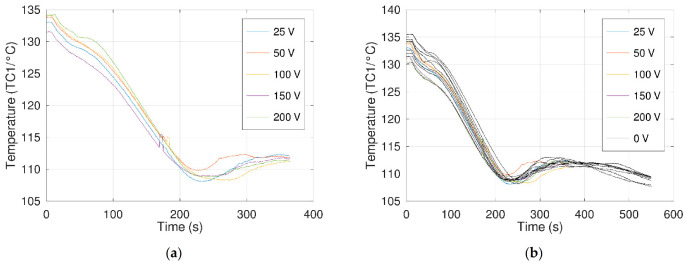
Thermograms obtained at the level of the electrode assembly (TC1) during the free cooling: (**a**) with electrical fields from 25 V to 200 V; (**b**) with electrical fields from 25 V to 200 V and superposition of the 9 thermograms obtained without an electric field (0 V).

The obtained results (Figure 8 and Table 6) showed a temperature of crystallization of 108.7 °C (standard deviation of 0.7 °C); thus, compared to the preliminary tests, no impact of the application of an electrical field was observed on the nucleation of erythritol. One can note that the crystallization is, as for the preliminary tests, always initiated at the level of electrodes and their support (Table 6).

Additional remarks can be made:

- During the tests with an E-field, the ammeter allowed us to measure electric currents from ca. 60 µA at 25 V to more than 1000 µA at 200 V. These amplitudes are consistent with the measurements of Jankowski and McCluskey. Moreover, data recorded via thermocouple TC1 presented a small peak when a potential difference was applied (Figure 8, at ca. 175 s), showing an interference with the potential difference measured between the thermocouple ends. These observations ensured that an electric current was flowing between the two electrodes.
- At 150 V, erythritol started to caramelize between the electrodes.
- At 200 V, a “dark cloud” linked to the degradation of erythritol was observed between the electrodes, an observation also reported by Jankowski and McCluskey but at 300 V [18]. This degradation at lower voltage can be explained by a small difference in the inter-electrode gaps of the two setups.

The strong differences between the results obtained in this work and those reported by Jankowski and McCluskey led to the formulation of various hypotheses. The first one, already mentioned in Section 3.1.2, is that the silver electrodes act as a thermal bridge, decreasing locally the temperature of erythritol to its crystallization temperature, resulting in an apparent reduction in the degree of supercooling. In previous work, it is not clear whether the entire electrode assembly was immersed during the preliminary tests (i.e., without an E-field) carried out by the authors. It is therefore possible that after introduction of the electrode assembly, they were able to observe a reduction in the degree of supercooling comparable to that measured in our work but due to a similar heat sink effect and not to the application of an electric field. In addition, a doubt remains regarding the impact of the purity of the sugar alcohol used: 99% in this work and food grade in the previous one. Finally, the complexity of the phenomena involved and the multiple possibilities of introducing bias can lead to different observations. This complexity was summarized by Beaupere et al. [9], underlining the difficulty of reproducing and interpreting the works dedicated to the study of the impact of E-fields on the supercooling of water. 

One can note that a solution, out of the scope of the present paper, based on local cooling (thermal bridge) of erythritol could be viable to promote its nucleation, given the remarkable repeatability observed and the relative simplicity of implementation in a storage system [6]. 

Nevertheless, our observations show that in any case, configuration 1 is not suitable to study the impact of electric fields on erythritol nucleation. Indeed, without the possibility of maintaining the sugar alcohol in a supercooled state because of the thermal bridge effect, this study becomes too delicate.

Considering the previous findings, configuration 2 should be more appropriate. In-deed, a higher amount of erythritol immersing almost all the electrode assembly should limit the thermal dissipation at the electrodes’ extremities. The results obtained are presented in the next section.

#### 3.2.2. Configuration 2

For the second configuration, the applied procedure can be described as follows:

1. A 30 mL beaker containing the electrode assembly and the sugar alcohol was heated in an oil bath until erythritol melted and thermocouple TC1 indicated a temperature of about 130 °C (~12 °C above the melting temperature of erythritol).
2. Using the thermal regulation of the hot plate, the oil bath was cooled down until TC1 indicated the desired temperature: 100, 95, or 90 °C in our tests. This temperature range was chosen in accordance with the results obtained by Duquesne et al. [26]. Indeed, at these temperatures, the crystal growth velocity of erythritol is maximum, which will facilitate its observation. One of the advantages of this procedure compared to that of configuration 1 is to avoid parasitic vibrations generated by the extraction of the beaker from the oil bath.
3. When the temperature according to TC1 stabilized (±0.5 °C), a potential difference was imposed to the electrodes. Different electrical solicitations were tested:
  - Solicitations from 5 to 60 s in DC at 10, 50, 100, 150, and 200 V
  - Solicitations of 5 to 60 s in AC at 150 V with a frequency of 15, 30, 60, 200, 500, and 1000 Hz
  - Pulse-type solicitations of ca. 15 kV using a piezoelectric element
4. The electrical stress was then interrupted using the manual switch or the power supply switch (“floating electrode” or not, respectively).
5. After an observation period of ca. 15 min, the hot plate was switched off to allow the free cooling of the setup.
6. Temperatures from thermocouples TC1, TC2, and TCbath were recorded during steps 3 to 5 until complete crystallization of the erythritol. During this time, the beaker was visually inspected to identify the initial crystallization location.
7. Steps 1 to 6 were repeated for each experiment.

**Figure 9:**
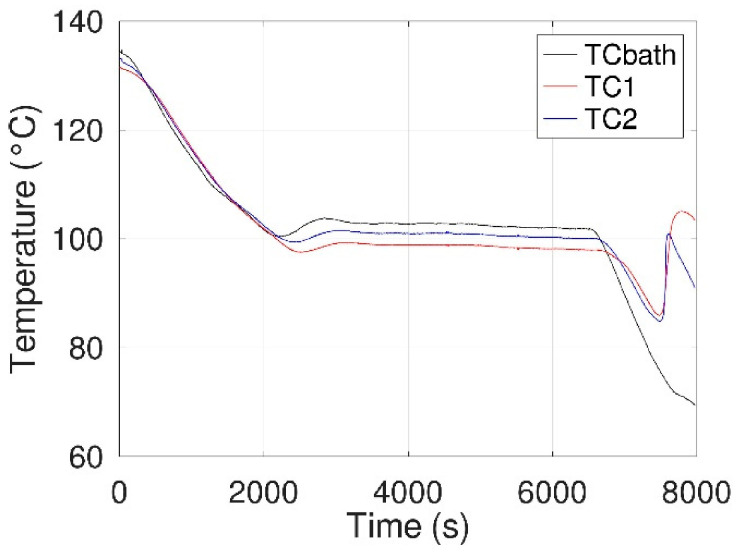
Thermograms (TC1, TC2, and TCbath) obtained at 99 °C (±0.5 °C) with an electrical field (200 V during 5 and 20 s).

As an example, Figure 9 shows one of the experiments, performed at ca. 100 °C, for two different electrical solicitations (200 V during 5 s at t = 4555 s and 200 V during 20 s at t = 5515 s) with an observation period of 15 min after each of them. It can be seen that no crystallization occurred following these solicitations, nor following the other tests carried out, i.e., the tests performed at different temperatures of supercooling and/or applied E-fields. 

During these tests, it was always possible to ensure that an electric current was flowing between the two electrodes: from 35 µA at 100 V to 380 µA at 500 V.

**Figure 10:**
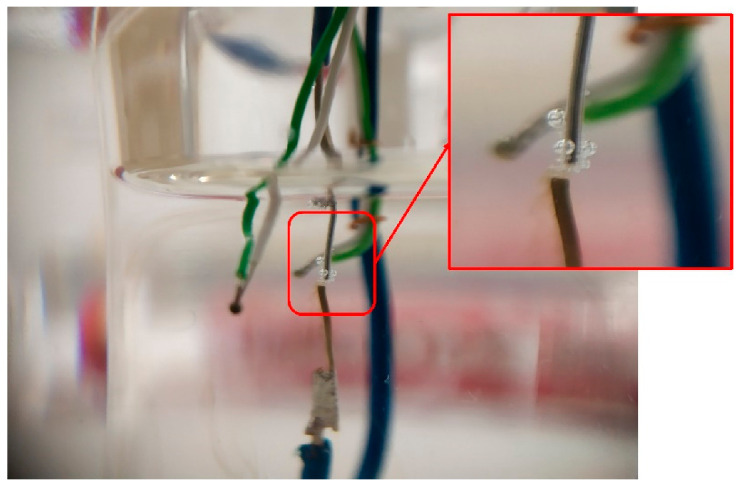
Bubble formation at 50 V during ca. 20 s.

In addition, one can note (Figure 10 and the corresponding video in Supplementary Materials) that high voltages and/or long times of exposure (ca. > 10 s) led to small bubble formation between the electrodes, their amount increasing with the voltage. 

**Figure 11:**
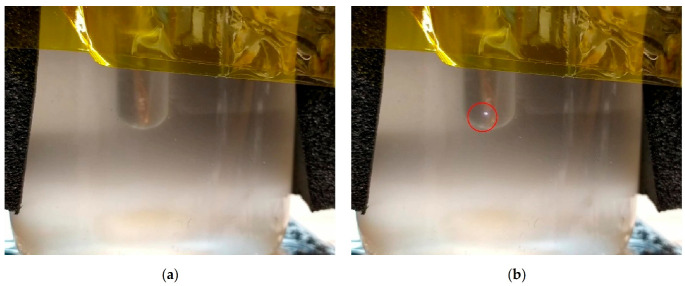
Pictures taken during the experiments at high voltage and short time of exposure: (**a**) before an electric pulse; (**b**) generation of an electrical arc (center of the red circle).

Additional experiments were also carried out using a piezoelectric element to produce an electrical arc (Figure 11). The different tests carried out under this high electrical field again led to the formation of bubbles but never promoted the crystallization of erythritol. 

Additional tests carried out with AC current at different frequencies, with electrodes of different materials (silver and copper), with or without floating electrode mode, led to similar observations, i.e., no electric-field-induced crystallization was observed, while bubbles were generated at the electrodes at high voltages and/or long times of exposure (ca. > 10 s).

This phenomenon of bubble generation has already been reported by Hozumi [22,23] and Shichiri [27] and may be related to hydrogen production at the level of the electrodes. However, unlike in the experiments performed on supercooled water by the latter, no im-pact was observed on the crystallization of erythritol in our experiments. It is likely that a local electrochemical process leads to decomposition of the sample, evidenced in both configurations by caramelization, or even the formation of a black cloud at high voltage, counterbalancing this potentially positive effect.

Finally, despite the improvement of the experimental setup and the multiple parameters tested, it was not possible at any time to show any effect of electric fields on the crystallization of erythritol.

## 4. Conclusions

In this experimental study, two configurations were tested to promote the crystallization of erythritol using an electric field. A wide spectrum of electrical solicitations was explored: direct and alternating currents at different frequencies and amplitudes, electrode materials (silver and copper) tested in floating mode or non-floating mode, and tests of long or short duration.

Configuration 1, devoted to the reproduction of the work of Jankowski and McCluskey to study the effect of E-fields on the same sugar alcohol (erythritol), was tested in the first step. Despite using a configuration as similar as possible to that described in this previous work, it was not possible to reproduce their results. Surprisingly, results obtained in our study exhibited a similar degree of supercooling, with higher repeatability, but without the use of an electric field. Furthermore, the use of different voltages on this polyol did not show any impact on these results either.

These differences between both studies led to the formulation of various hypotheses:

- The electrodes/thermocouples act as thermal bridges, decreasing locally the temperature of erythritol to its crystallization temperature, leading to an apparent reduction in the degree of supercooling. It is possible that a similar effect was observed in the previous work.
- The purity of erythritol may have a significant impact on the observed results (99% in this work, food grade in the previous one)
- The complexity of the phenomena involved during E-field-induced crystallization experiments and the multiple possibilities for introducing bias, already highlighted in different papers, can explain the difficulty of reproducing and interpreting the authors’ results.

Considering the previous findings, it was decided to modify the experimental setup by limiting the heat sink phenomenon induced by the electrodes suspended above erythritol. Thus, in configuration 2, a higher amount of polyol was tested to better immerse the modified electrode assembly, allowing us to perform the planned tests under the desired conditions, i.e., without a drastic reduction in the degree of supercooling before any electrical stress. 

During these tests, it was always possible to ensure that an electric current was flowing between the electrodes by means of an ammeter. In addition, for high-voltage and/or long-duration experiments, bubble generation was observed at the level of the electrodes, which could promote the crystallization of the sugar alcohol. However, a local electrochemical process, which can lead to the decomposition of the sample (caramelization of erythritol or even the formation of a black cloud at high voltage), seems to counterbalance this potentially positive effect. 

Finally, despite the large number of parameters tested, it has never been possible to influence the crystallization of the erythritol using electric fields.

Due to the complexity of the involved phenomenon and the number of influencing parameters, such as the nature and configuration of the electrodes, their gap or their shape, and the nature and purity of the sample, further experiments and theoretical studies are necessary on this topic.

## Figures and Tables

**Table 1:** Main properties of erythritol [19,20].

Material	CAS Number	Formula	Phase Change Temperature (°C)	Mass-Specific Enthalpy (kJ/kg)	Degree of Supercooling (°C)
Erythritol	149-32-6	C_4_H_10_O_4_	117–120	315–379	Up to 65

**Table 2:** Description of the electrodes used in the two configurations.

Configuration	Material	Type	Dimensions (mm)	Gap (mm)
1	Silver	Wire	0.4 Ø	0.35
2	Silver Copper	Wire	0.4 Ø 0.2 Ø	1.5

**Table 3:** Erythritol properties measured by DSC.

Cycle	Mass-Specific Enthalpy (kJ/kg)	Melting Temperature (°C)	Temperature of Crystallization (°C)	Degree of Supercooling (°C)
1	317.0	118.4	58.5	60.0
2	319.7	118.2	53.4	64.8
3	319.0	118.4	53.2	65.2
Average	318.6	118.3	55.0	63.3
Standard deviation	1.4	0.1	3.0	2.9

**Table 4:** Preliminary tests without applying a current.

Trial	Initial Nucleation Location	Local Temperature (°C)
PRE01	Electrode assembly	107.9
PRE02	Electrode assembly	108.6
PRE03	Electrode assembly	108.9
PRE04	Electrode assembly	108.8
PRE05	Electrode assembly	108.8
PRE06	Electrode assembly	109.0
PRE07	Electrode assembly	108.9
PRE08	Electrode assembly	107.6
PRE09	Electrode assembly	108.2

**Table 5:** Preliminary tests without applying a current.

Trial	Initial Nucleation Location	Local Temperature (°C)
PRE01	Bottom of the electrode assembly	68.6
PRE02	Top of the electrode assembly	65.7
PRE03	Center of the electrode assembly	75.5

**Table 6:** Obtained results applying a current.

Trial	Voltage (V)	Initial Nucleation Location	Local Temperature (°C)
V01	25	Electrode assembly	108.1
V02	50	Electrode assembly	109.8
V03	100	Electrode assembly	108.2
V04	150	Electrode assembly	108.9
V05	200	Electrode assembly	108.5

## Data Availability

Data is contained within the article or Supplementary Materials.

## Supplementary Materials

The following are available online at https://www.mdpi.com/article/10.3390/ma14175110/s1, Video S1: VID_50V_Conf2.
